# Sample Size Determination for Individual Bioequivalence Inference

**DOI:** 10.1371/journal.pone.0109746

**Published:** 2014-10-13

**Authors:** Chieh Chiang, Chin-Fu Hsiao, Jen-Pei Liu

**Affiliations:** 1 Division of Biometry, Department of Agronomy, National Taiwan University, Taipei, Taiwan; 2 Institute of Epidemiology and Preventive Medicine, National Taiwan University, Taipei, Taiwan; 3 Division of Biostatistics and Bioinformatics, Institute of Population Health Sciences, National Health Research Institutes, Zhunan, Taiwan; National Institute of Environmental and Health Sciences, United States of America

## Abstract

Statistical criterion for evaluation of individual bioequivalence (IBE) between generic and innovative products often involves a function of the second moments of normal distributions. Under replicated crossover designs, the aggregate criterion for IBE proposed by the guidance of the U.S. Food and Drug Administration (FDA) contains the squared mean difference, variance of subject-by-formulation interaction, and the difference in within-subject variances between the generic and innovative products. The upper confidence bound for the linearized form of the criterion derived by the modified large sample (MLS) method is proposed in the 2001 U.S. FDA guidance as a testing procedure for evaluation of IBE. Due to the complexity of the power function for the criterion based on the second moments, literature on sample size determination for the inference of IBE is scarce. Under the two-sequence and four-period crossover design, we derive the asymptotic distribution of the upper confidence bound of the linearized criterion. Hence the asymptotic power can be derived for sample size determination for evaluation of IBE. Results of numerical studies are reported. Discussion of sample size determination for evaluation of IBE based on the aggregate criterion of the second moments in practical applications is provided.

## Introduction

The traditional criterion for evaluation and approval of small-molecular chemical generic drug products is based on average bioequivalence (ABE). ([1] – [4]) On the other hand, biosimilar drugs and most of targeted drugs are biological products which are fundamentally different from traditional small-molecular chemical generic drugs in size, functional structure, physiochemical properties, impurities, immunogenicity and manufacturing processes. However, ABE considers only equivalence between population means and completely ignores the variability of the drug products and that of formulation effects between patients. Therefore, ABE is not an adequate criterion for evaluation of the generic copies of targeted drugs and biosimilar drug products. On the other hand, individual bioequivalence (IBE) simultaneously takes differences in population means, subject-by-formulation interaction, and within-subject variability into account. ([1], [4]) As a result, IBE may be more appropriate for evaluation of generic targeted drugs and biosimilar products. ([5], [6]).

The U.S. Food and Drug Administration (FDA) Guidance for Industry “*Statistical Approaches to Establishing Bioequivalence”* recommends replicated crossover designs for IBE studies [1]. The linearized criterion for IBE evaluation suggested in the U.S. FDA guidance is the linear combination of the squared mean difference, variance of subject-by-formulation interaction, and the difference in within-subject variances between the generic and innovative products. The U.S. FDA guideline proposes the upper confidence bound for the linearized form of the IBE criterion derived by the modified large sample (MLS) method as a testing procedure for evaluation of IBE. In other words, generic and innovative products are claimed to be IBE if the MLS 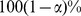 upper confidence bound of the linearized criterion is less than zero. Despite a vast literature on various methodologies for evaluation of IBE, literature on sample size determination for evaluation of IBE is scarce. Under the two-sequence and four period (2×4) crossover design, we derive the asymptotic distribution of the MLS 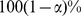 upper confidence bound and the asymptotic power for sample size determination for the IBE evaluation. Our approach is to determine the sample size to provide the asymptotic power for which the MLS 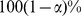 upper confidence bound for the IBE criterion smaller than zero is greater than 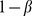.

In the next section, the method for construction of the MLS upper confidence bound for the IBE criterion for the 2×4 crossover design is reviewed. Our proposed methods of sample size determination for IBE evaluation based on the asymptotic distribution of the MLS upper confidence bound are then presented. The results of numerical studies, including numerical examples and simulation studies, are provided in the next section. Numerical examples illustrate applications of our proposed method in practical scenarios. Simulation studies were conducted to investigate the impact of magnitudes of means differences, variance of subject-by-formulation interaction, and within-subject variances on sample sizes. In addition, empirical powers obtained from simulation studies are compared with the asymptotic powers to examine whether the sample sizes determined by our proposed methods can provide sufficient power. Discussion and final remarks are given in the last section.

## Methods

### Criterion for Individual Bioequivalence

In what follows, unless otherwise specified, all parameters and statistics are on the log-scale. Let 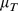 and 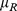 be the mean for test (generic product) and reference (innovative product) formulations, respectively. In addition, 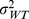 and 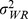 denote the within-subject variance for the test and reference formulation, respectively, and let 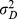 be the variance of the subject-by-formulation interaction. The IBE criterion [1], [4], [7] is defined as
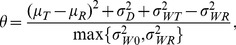
(1)where 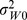 is the specified constant within-subject variance, which the U.S. FDA guidance suggests that it be set at 0.04 [1]. Based on the IBE criterion given in Equation (1), the null hypothesis of non-IBE and the alternative hypothesis of IBE are respectively given as
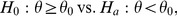
(2)where 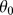 is the upper limit of the IBE criterion, which is set as 2.4948 in the U.S. FDA guidance [1].

Hyslop et al [5] suggested the following linearized IBE criterion for assessment of IBE:
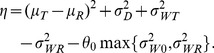
(3)


To avoid direct estimation of 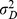, the linearized IBE criterion in Equation (3) can be re-expressed as [8].
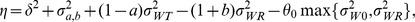
where 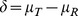, 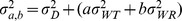 for 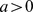 and 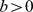. For the 2×4 crossover design, a = b = 0.5. Hence the linearized IBE criterion becomes



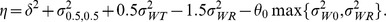



When 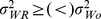, 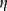 is referred to as the linearized reference (constant)-scaled criterion. Consequently, the IBE hypotheses given in Equation (2) can be reformulated using the linearized criterion as follows:
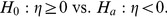
(4)


### Upper Confidence Bound by the Modified Large Sample (MLS) Method

Under the 2×4 crossover design (TRTR, RTRT) given in File S1, a MLS 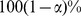 upper confidence bound [7] is given as
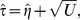
(5)where 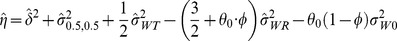, and
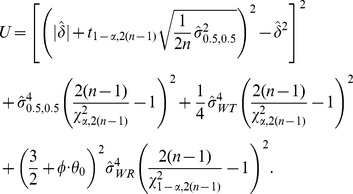
(6)with 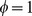 if 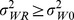 and 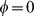 if 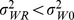. Here *n* is the sample size (the number of subjects) per sequence, and 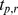 and 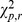 are the 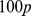
th percentiles of the central t and central chi-square distributions, respectively, with 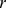 degrees of freedom. Estimators 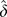, 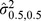, 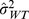, 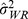 and derivation of the MLS 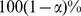 upper confidence bound for the linearized IBE criterion are given in File S1. The null hypothesis is rejected and the IBE is concluded at the 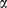 significance level if the MLS 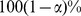 upper confidence bound given in Equation (5) is less than zero.

### Sample Size Determination

By the delta method, 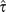 is asymptotically normal with mean 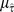 and variance 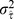. Proof of the asymptotic normality of 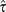 and derivations of 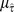 and 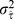 are given in File S2 [8], [9]. Let 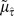 and 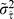 be some specified values of 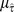 and 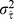 respectively in the alternative hypothesis. An asymptotic power based on the MLS upper confidence bound using the normal distribution can be computed as
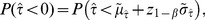
(7)where 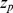 is the 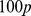
th percentile of standard normal distribution. Based on the mean value theorem, the derivatives of 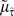 and 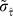 with respect to 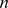 for a small constant 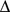 are given as



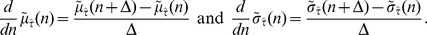



It follows that the smallest 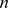 can be derived as 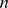 converges at the (*l*+1)th iteration, where
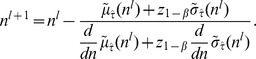



Since Equation (7) is derived directly from the asymptotic power, there exists only one solution for sample size determination with respect to the required power. Equation (7) can be evaluated by the numerical method. File S3 provides a SAS macro in PROC NLP (nonlinear programming) by the quasi-Newton method. This SAS macro is flexible to allow users to specify the significance level, the required power, the upper IBE equivalence limit, 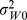, and the mean difference, the variance of subject-by-formulation interaction and the within-subject variances for the test and reference formulations.

### Simulation Setup

The first objective of simulation studies is to determine the sample sizes for the nominal 80% power at the 5% significance level under different specifications for various combinations of parameters under the 2×4 crossover design (TRTR, RTRT). The second objective is to investigate the impact of magnitudes of means differences, variance of subject-by-formulation interaction, and within-subject variances on sample size The third objective is to compare the empirical power obtained from simulation studies with the asymptotic power obtained by Equation (7) and the nominal power of 80%. Because there are four parameters, a four-factor factorial simulation study with three levels for each factor was employed. Simulation studies were performed separately for the constant-scaled criterion and reference-scaled criterion. Four levels of the within-subject reference variance were used for the reference-scaled criterion. It follows that 3×3×3×3 and 3×3×4×3 factorial simulation studies were employed in simulation studies for the constant-scaled and reference-scaled criteria, respectively. The values of mean difference are set to be 0, 0.05, and 0.1. For the constant-scaled criterion, the magnitudes of the reference within-subject variance are specified to be 0.01, 0.02, and 0.03. They are 0.04, 0.09, 0.16, and 0.25 for the reference-scaled criterion. In order to investigate the impact of an increasing or reduction of the test within-subject variance on the sample size, the differences in the magnitude of the within-subject variance between the test and reference formulations are set to be −0.005, 0, and 0.005 for the constant-scaled criterion and −0.02, 0, and 0.02 for the reference-scaled criterion. The values of the variance of the subject-by-formulation interaction were selected in proportion to the magnitude of the within-subject variances. They are set to be 0.0001, 0.001, and 0.0225.

Table 1 provides the specifications of various combinations of the four parameters. The sample size for each of a total of 189 combinations given in Table 1 was determined by the proposed method. Under the model of the 2×4 crossover design in Equation (S1.1) in File S1, 10,000 random samples are generated according to the sample size obtained by the proposed method and the specification of the magnitudes for a particular combination of parameters. The MLS 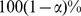 upper confidence bound for the IBE linearized criterion is then computed for each generated random sample, according to Equation (5). The empirical power is calculated as the proportion of the random samples with the MLS 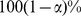 upper confidence bounds smaller than zero. For 10,000 random samples, it implies that the 95% of the empirical powers would be greater than 0.7934 if the sample size obtained by the proposed method can provide sufficient power with respect to the nominal power of 80%.

**Table 1:** Specifications of parameters for simulation studies.

Parameters	Specifications
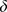	0, 0.05, 0.1
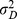	0.0001, 0.01, 0.0225
Constant-scaled criterion	
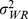	0.01, 0.02, 0.03
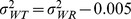	0.005, 0.015, 0.025
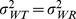	0.01, 0.02, 0.03
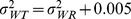	0.015, 0.025, 0.035
Reference-scaled criterion	
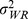	0.04, 0.09, 0.16, 0.25
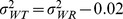	0.02, 0.07, 0.14, 0.23
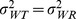	0.04, 0.09, 0.16, 0.25
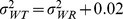	0.06, 0.11, 0.18, 0.27

## Results

### Numerical Examples

For the purpose of illustration, examples of sample size determination under the 2×4 crossover design (TRTR, RTRT) for both the linearized constant-scaled criterion and reference-scaled criterion are provided. Under the linearized constant-scaled criterion, the specifications of the parameters for the sample size are 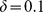, 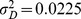 and 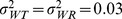. It follows that
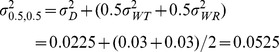



and
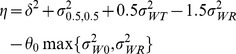


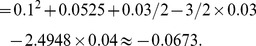



Using the SAS macro given in File S3, the sample size for the nominal power of 80% at the 5% significance level is 16 subjects per sequence. Since the asymptotic mean in Equation (S2.5) in File S2 is 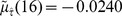 and variance in Equation (S2.6) of File S2 is 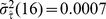, the corresponding asymptotic power in Equation (7) is given as
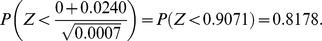



Suppose that both 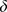 and 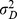 are kept the same and both 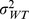 and 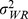 increase to 0.05. Since 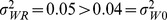, the linearized reference-scaled criterion is used. It follows that
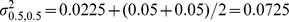



and
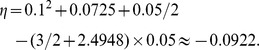



Under this scenario, the sample size is 29 subjects per sequence for the nominal power of 80% at the 5% significance level with 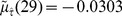, 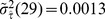, and an asymptotic power
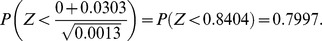



If 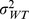 increases to 0.07 and 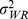 remains as 0.05, the sample size per sequence is increased to 53 with 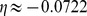, 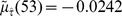, 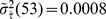 and an asymptotic power of 0.8039. However if 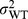 decreases to 0.03 and 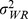 remains the same, the sample size is reduced to 18 subjects per sequence with 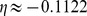, 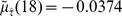, 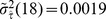 and the corresponding asymptotic power 0.8046. Therefore, if the test formulation has a better quality by reducing the within-subject variability, fewer subjects are required for evaluation of IBE under the 2×4 crossover design.

### Results of Simulation Studies

**Figure 1:**
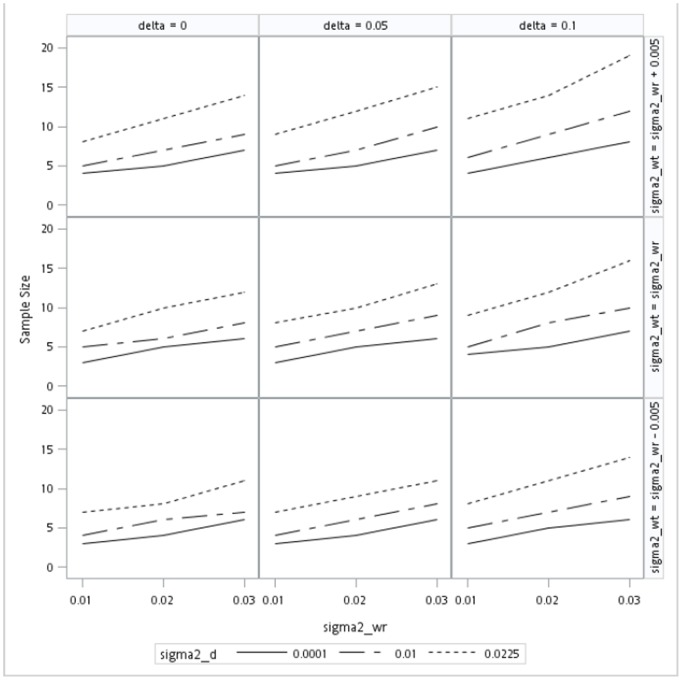
Sample size per sequence for responses with *α* = 0.05 and power = 0.8 under constant-scaled criterion.

Figure 1 provides a graphic 3×3 presentation of the sample sizes of all 81 combinations considered for the linearized constant-scaled criterion. The three vertical panels are arranged by the mean difference in an ascending order from left to right. The three horizontal panels are presented by the within-subject variance of the test formulation in a descending order from top to bottom. For each of the nine cells, the vertical axis is the sample size, while the horizontal axis is the within-subject variance of the reference formulation. Three lines within each cell represent the sample sizes obtained from different values of the variance of subject-by-formulation interaction.

Figure 1 and Table S1 reveal that sample size ranges from 3 to 19 subjects per sequence for all 81 combinations considered under the linearized constant-scaled criterion and the 2×4 crossover design. However, the linearized constant-scaled criterion in Equation (3) is an increasing function of mean difference, variance of the subject-by-formulation interaction, and the difference in within-subject variances between the test and reference formulations. Figure 1 reveals that the sample size is also an increasing function of mean difference and variance of the subject-by-formulation interaction. For our simulation studies, the difference in within-subject variances between the test and reference formulations is set to be −0.005, 0, and 0.005. It follows that the linearized constant-scaled criterion is a function only of mean difference and the variance of the subject-by-formulation as long as the difference in within-subject variances between the test and reference formulations is a constant. In other words, since 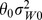 is a constant, 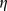, as shown in Table S1, is also a constant for any fixed specification of 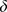 and 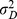. However, Figure 1 also reveals that sample size increases as the reference within-subject variance 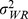 increases. This phenomenon may be due to the fact that the upper confidence bound in Equation (5) is an increasing function of the estimated within-subject variance of the reference formulation 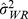. On the other hand, a reduction of sample size can be achieved if the within-subject variance of the test formulation is smaller than that of the reference formulation. Otherwise, more subjects are required.

**Figure 2:**
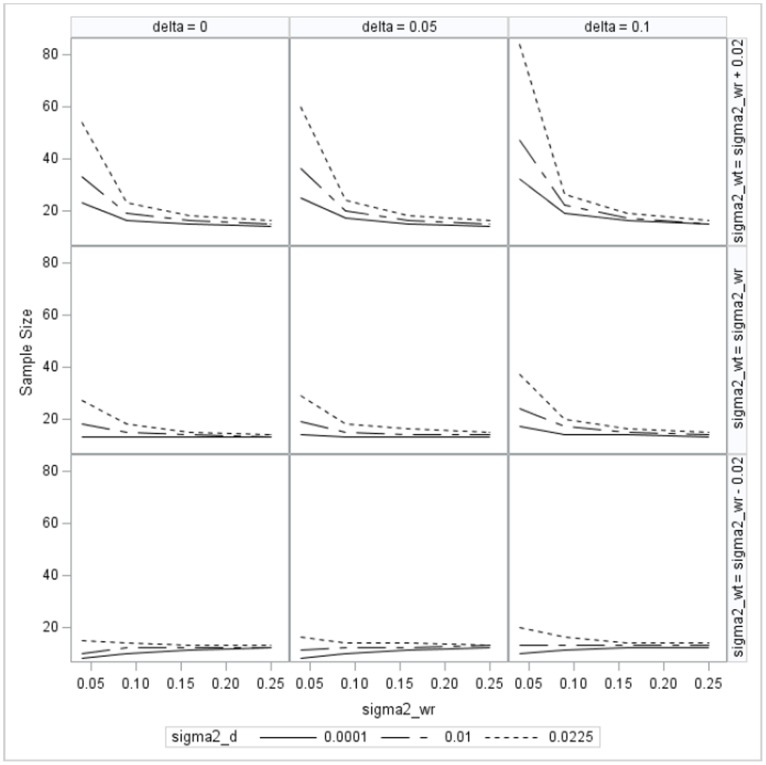
Sample size per sequence for responses with *α* = 0.05 and power = 0.8 under reference-scaled criterion.

Sample sizes of all 108 combinations for the linearized reference-scaled criterion are also presented in a 3×3 graphical display in Figure 2. Sample sizes given in Figure 2 and Table S2 for the linearized reference-scaled criterion range from 8 to 84 per sequence. As a result, the range of the sample sizes for the linearized reference-scaled criterion is much wider that those of the linearized constant-scaled criterion because 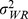 is confined to a narrow range between 0 and 0.04 for the constant-scaled criterion. Similar to the results of the linearized constant-scaled criterion, the sample size for the linearized reference-scaled criterion is an increasing function of mean difference and variance of the subject-by-formulation interaction, and fewer subjects are needed when the within-subject variance of the test formulation is smaller than that of the reference formulation.

However, a striking difference in the trend of sample sizes between Figure 1 and Figure 2 is that except for the specification when 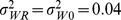, the sample size for the linearized reference-scaled criterion is a deceasing function of the within-subject variance of the reference formulation as depicted in Figure 2. This is due to the fact that 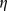 is a decreasing function of 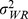. Except for the specification of 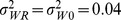, Table S2 shows that 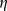 deceases from −0.2044 to −0.6436. On the other hand, the range of 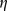 for the linearized constant-scaled criterion is only from −0.0623 to −0.1047, as given in Table S1. For any fixed specification of 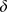 and 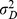, the maximum of 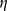 occurs when 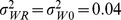. As a result, as shown in Figures 1 and 2 and Tables S1 and S2, when 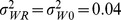, the required sample size per sequence is the largest for any fixed specification of 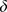 and 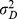.

Tables S1 and S2 also provide the asymptotic and empirical powers for a total of 189 combinations. Only 2 of the 189 empirical powers (1.05%) are below 0.7934. This demonstrates that with respect to the nominal power of 80%, the sample size obtained by our proposed method can provide sufficient power for evaluation of IBE under the 2×4 crossover design. Because of a narrow range of 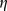 and 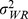, 60 of 81 sample sizes (84.5%) for the linearized constant-scaled criterion are smaller than 10. Due to the discrete nature of the sample size, both asymptotic and empirical powers are from 0.8107 to 0.9598, which are larger than the nominal power of 80%. On the other hand, for the linearized reference-scaled criterion, only 2 out of 108 sample sizes (1.85%) are below 10. Consequently, the range of 108 empirical powers is from 0.7905 to 0.8289. It follows that with respect to a nominal power of 80%, the sample size obtained by the proposed method for the linearized reference-scaled criterion provides neither insufficient nor excessive power. Moreover, the maximum of absolute differences between empirical power and asymptotical power is 0.0258. In addition, only 29 of 189 absolute differences (15.3%) are greater than 0.01. This shows that the asymptotic power used for the sample size determination by the proposed method is quite accurate, as verified by the empirical power obtained by simulation studies.

## Discussion

Although the upper confidence bound constructed by the MLS method for the linearized criterion has been used for evaluation of IBE, literature on analytical determination of sample size is scarce. We showed that the MLS upper confidence bound converges asymptotically to a normal distribution. Hence, we propose an analytical procedure for sample size determination for evaluation of IBE based on the approximate power derived from the asymptotic normal distribution of the MLS upper confidence bound of the linearized criterion under the 2×4 crossover design. Extensive simulation studies show that the sample sizes obtained by our proposed method can provide sufficient and yet not excessive power. In addition, the results of simulation studies also reveal that the approximation of the asymptotic power is quite accurate, as verified by the empirical power. Simulation studies also investigated the impact of magnitudes of the four parameters on sample sizes. Our numerical studies found that smaller sample sizes can be obtained if the within-subject variance of the reference formulation is less than 0.04 or the within-subject variance of the test formulation is smaller than that of the reference formulation.

For any fixed specification of 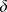, 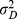 and 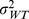, 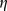 is a decreasing function of 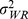. However, the decreasing rate for the linearized constant-scaled criterion is −1 in a narrow range from 0 to 0.04 with a constant constraint of 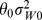. On the other hand, 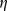 for the linearized reference-scaled criterion has a much faster decreasing rate of 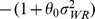. Therefore, the maximum sample size for evaluation of IBE occurs when the within-subject variance of the reference formulation is at the boundary point of 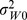.

The objective of the specified constant within-subject variance 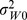 in the constant-scaled criterion is to avoid a larger upper confidence bound of the IBE criterion when the reference product exhibits extremely small within-subject variability to prevent approval of any generic products. Sample sizes of all 81 combinations for the linearized constant-scaled criterion are smaller than 20 per sequence. This demonstrates that the IBE evaluation by the constant-scaled criterion can be accomplished with a reasonable sample size with respect to a nominal power of 80% at the 5% significance level if the within-subject variance of the reference formulation is smaller than 0.04. On the other hand, when 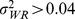, the sample size is a decreasing function of 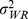. This contradicts the usual intuition that a larger variability requires a larger sample size.

The proposed method can also be easily adapted to other crossover designs such as the 2×3 crossover design (TRT, RTR). Table S3 compares the sample sizes required between the 2×3 crossover design (TRT, RTR) and the 2×4 crossover design (TRTR, RTRT) for a nominal power of 80% at the 5% significance level. Table S3 reveals that the number of subjects required for the 2×3 crossover design increases from 71% to 107% over that required by the 2×4 crossover design. Each subject in the 2×3 crossover design yields 3 observations per subject as compared to 4 observations per subject by the 2×4 crossover designs. However, the total number of observations for the 2×3 crossover design is still greater than that of the 2×4 crossover design. Therefore, although the duration of the 2×3 crossover design is shorter, the 2×4 crossover design is still more efficient for evaluation of IBE than the 2×3 crossover design.

In practice, one of the key issues is selection of the reference-scaled criterion or constant-scaled criterion for evaluation of IBE. Three methods have been proposed. The first method is referred as to the estimation method (EST) suggested by Hyslop et al. [7]. The estimation method recommends using the reference-scaled criterion or constant-referenced criterion according to 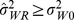 or 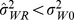. The second method is the test method (TEST) which tests the hypothesis of 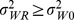 vs. 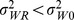 to decide which criterion should be used. [8] If 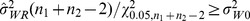, then the reference-scaled criterion should be used; otherwise the constant-scaled criterion should be used. The third method (OPT) assumes that we know whether 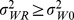. [10] Chow et al. [10] conducted simulation studies to compare the three methods. When 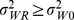, all the three methods perform equally well in controlling the type I error rate. However, when 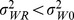, the tests using the estimation method for choosing the reference-scaled criterion or constant-scaled criterion slightly inflate the type I error rate but only up to 0.06. On the other hand, the test using the test method is conservative when 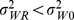. When 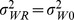, the test method performs slightly better than the estimation method.

We also conducted additional simulation studies to compare empirical powers of the three methods when 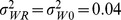. The results are provided in Table S4. Most of differences in empirical powers between the three methods and the asymptotic powers are in the second or third decimal point. Except for only two cases, the difference between the empirical power by the estimation method and the asymptotic power does not exceed 10%. From Table S4, we reconfirm that the test method should be used when 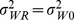 because, except for one case, all differences are in the third decimal point. In summary, when 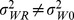, the estimation method should be used to select the criterion. On other hand, when 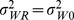 or 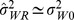, the test method should be used to choose the reference-scaled criterion or constant-scaled criterion.

## Supporting Information

Table S1 **Sample size per sequence, asymptotical power, and empirical power for the linearized constant-scaled criterion with respect to a nominal power of 80% at the 5% significance level.**
(DOC)

Table S2 **Sample size per sequence, asymptotical power, and empirical power for the linearized reference-scaled criterion with respect to a nominal power of 80% at the 5% significance level.**
(DOC)

Table S3 **Comparison of sample sizes required between the 2×3 and 2×4 crossover designs with respect to a nominal power of 80% at the 5% significance level.**
(DOC)

Table S4
**Comparison between different methods for determining the scaled criterion with respect to a nominal power of 80% at the 5% significance level, where 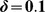, 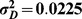, and 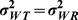.**
(DOC)

File S1
**Derivation of the 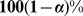 upper confidence bound under 2×4 crossover design by the modified large sample method.**
(DOC)

File S2 **Derivation of the asymptotic normality of the upper confidence bound.**
(DOC)

File S3 **SAS macro code.**
(DOC)
