# Supplementary material for: Sample Size Determination for Individual Bioequivalence Inference
Source: PLoS One. 2014 Oct 13;9(10):e109746. doi: 10.1371/journal.pone.0109746 (PMC4195669; doi:10.1371/journal.pone.0109746)
Supplement: Table S1 — Sample size per sequence, asymptotical power, and empirical power for the linearized constant-scaled criterion with respect to a nominal power of 80% at the 5% significance level. (DOC) [file pone.0109746.s001.doc]

Table S1 Sample size per sequence, asymptotical power, and empirical power for the linearized constant-scaled criterion with respect to a nominal power of 80% at the 5% significance level

| $\delta$ | $\sigma_{D}^{2}$ | $\sigma_{WT}^{2}$ | $\sigma_{WR}^{2}$ | $\eta$ | $n$ | Asymptotic Power | Empirical Power | Difference  in Power |
| --- | --- | --- | --- | --- | --- | --- | --- | --- |
| 0 | 0.0001 | 0.01 | 0.01 | -0.0997 | 3 | 0.8581 | 0.8526 | 0.0055 |
| 0.05 |  |  |  | -0.0972 | 3 | 0.8316 | 0.8343 | -0.0027 |
| 0.1 |  |  |  | -0.0897 | 4 | 0.9805 | 0.9598 | 0.0207 |
| 0 | 0.01 | 0.01 | 0.01 | -0.0898 | 5 | 0.9297 | 0.9152 | 0.0145 |
| 0.05 |  |  |  | -0.0873 | 5 | 0.9045 | 0.8929 | 0.0116 |
| 0.1 |  |  |  | -0.0798 | 5 | 0.8162 | 0.8275 | -0.0113 |
| 0 | 0.0225 | 0.01 | 0.01 | -0.0773 | 7 | 0.8205 | 0.8341 | -0.0136 |
| 0.05 |  |  |  | -0.0748 | 8 | 0.8675 | 0.8653 | 0.0022 |
| 0.1 |  |  |  | -0.0673 | 9 | 0.8263 | 0.8348 | -0.0085 |
| 0 | 0.0001 | 0.02 | 0.02 | -0.0997 | 5 | 0.9415 | 0.9229 | 0.0186 |
| 0.05 |  |  |  | -0.0972 | 5 | 0.9208 | 0.9132 | 0.0076 |
| 0.1 |  |  |  | -0.0897 | 5 | 0.8465 | 0.8434 | 0.0031 |
| 0 | 0.01 | 0.02 | 0.02 | -0.0898 | 6 | 0.8257 | 0.8267 | -0.0010 |
| 0.05 |  |  |  | -0.0873 | 7 | 0.8924 | 0.8840 | 0.0084 |
| 0.1 |  |  |  | -0.0798 | 8 | 0.8780 | 0.8737 | 0.0043 |
| 0 | 0.0225 | 0.02 | 0.02 | -0.0773 | 10 | 0.8610 | 0.8631 | -0.0021 |
| 0.05 |  |  |  | -0.0748 | 10 | 0.8234 | 0.8260 | -0.0026 |
| 0.1 |  |  |  | -0.0673 | 12 | 0.8120 | 0.8145 | -0.0025 |
| 0 | 0.0001 | 0.03 | 0.03 | -0.0997 | 6 | 0.8482 | 0.8472 | 0.0010 |
| 0.05 |  |  |  | -0.0972 | 6 | 0.8183 | 0.8289 | -0.0106 |
| 0.1 |  |  |  | -0.0897 | 7 | 0.8329 | 0.8350 | -0.0021 |
| 0 | 0.01 | 0.03 | 0.03 | -0.0898 | 8 | 0.8248 | 0.8282 | -0.0034 |
| 0.05 |  |  |  | -0.0873 | 9 | 0.8611 | 0.8536 | 0.0075 |
| 0.1 |  |  |  | -0.0798 | 10 | 0.8248 | 0.8297 | -0.0049 |
| 0 | 0.0225 | 0.03 | 0.03 | -0.0773 | 12 | 0.8137 | 0.8166 | -0.0029 |
| 0.05 |  |  |  | -0.0748 | 13 | 0.8193 | 0.8262 | -0.0069 |
| 0.1 |  |  |  | -0.0673 | 16 | 0.8149 | 0.8200 | -0.0051 |

Table S1 Sample size per sequence, asymptotical power, and empirical power for the linearized constant-scaled criterion with respect to a nominal power of 80% at the 5% significance level (continued)

| $\delta$ | $\sigma_{D}^{2}$ | $\sigma_{WT}^{2}$ | $\sigma_{WR}^{2}$ | $\eta$ | $n$ | Asymptotic Power | Empirical Power | Difference  in Power |
| --- | --- | --- | --- | --- | --- | --- | --- | --- |
| 0 | 0.0001 | 0.015 | 0.01 | -0.0947 | 4 | 0.9682 | 0.9424 | 0.0258 |
| 0.05 |  |  |  | -0.0922 | 4 | 0.9527 | 0.9272 | 0.0255 |
| 0.1 |  |  |  | -0.0847 | 4 | 0.8895 | 0.8767 | 0.0128 |
| 0 | 0.01 | 0.015 | 0.01 | -0.0848 | 5 | 0.8375 | 0.8450 | -0.0075 |
| 0.05 |  |  |  | -0.0823 | 5 | 0.8017 | 0.8101 | -0.0084 |
| 0.1 |  |  |  | -0.0748 | 6 | 0.8365 | 0.8491 | -0.0126 |
| 0 | 0.0225 | 0.015 | 0.01 | -0.0723 | 8 | 0.8185 | 0.8300 | -0.0115 |
| 0.05 |  |  |  | -0.0698 | 9 | 0.8505 | 0.8555 | -0.0050 |
| 0.1 |  |  |  | -0.0623 | 11 | 0.8438 | 0.8410 | 0.0028 |
| 0 | 0.0001 | 0.025 | 0.02 | -0.0947 | 5 | 0.8581 | 0.8544 | 0.0037 |
| 0.05 |  |  |  | -0.0922 | 5 | 0.8272 | 0.8259 | 0.0013 |
| 0.1 |  |  |  | -0.0847 | 6 | 0.8677 | 0.8609 | 0.0068 |
| 0 | 0.01 | 0.025 | 0.02 | -0.0848 | 7 | 0.8444 | 0.8491 | -0.0047 |
| 0.05 |  |  |  | -0.0823 | 7 | 0.8085 | 0.8204 | -0.0119 |
| 0.1 |  |  |  | -0.0748 | 9 | 0.8587 | 0.8585 | 0.0002 |
| 0 | 0.0225 | 0.025 | 0.02 | -0.0723 | 11 | 0.8360 | 0.8394 | -0.0034 |
| 0.05 |  |  |  | -0.0698 | 12 | 0.8435 | 0.8483 | -0.0048 |
| 0.1 |  |  |  | -0.0623 | 14 | 0.8057 | 0.8105 | -0.0048 |
| 0 | 0.0001 | 0.035 | 0.03 | -0.0947 | 7 | 0.8675 | 0.8633 | 0.0042 |
| 0.05 |  |  |  | -0.0922 | 7 | 0.8373 | 0.8420 | 0.0047 |
| 0.1 |  |  |  | -0.0847 | 8 | 0.8282 | 0.8256 | 0.0026 |
| 0 | 0.01 | 0.035 | 0.03 | -0.0848 | 9 | 0.8195 | 0.8267 | -0.0072 |
| 0.05 |  |  |  | -0.0823 | 10 | 0.8455 | 0.8445 | 0.0010 |
| 0.1 |  |  |  | -0.0748 | 12 | 0.8419 | 0.8487 | -0.0068 |
| 0 | 0.0225 | 0.035 | 0.03 | -0.0723 | 14 | 0.8214 | 0.8259 | -0.0045 |
| 0.05 |  |  |  | -0.0698 | 15 | 0.8176 | 0.8195 | -0.0019 |
| 0.1 |  |  |  | -0.0623 | 19 | 0.8175 | 0.8206 | -0.0031 |

Table S1. Sample size per sequence, asymptotical power, and empirical power for the linearized constant-scaled criterion with respect to a nominal power of 80% at the 5% significance level (continued)

| $\delta$ | $\sigma_{D}^{2}$ | $\sigma_{WT}^{2}$ | $\sigma_{WR}^{2}$ | $\eta$ | $n$ | Asymptotic Power | Empirical Power | Difference  in Power |
| --- | --- | --- | --- | --- | --- | --- | --- | --- |
| 0 | 0.0001 | 0.005 | 0.01 | -0.1047 | 3 | 0.9803 | 0.9548 | 0.0255 |
| 0.05 |  |  |  | -0.1022 | 3 | 0.9708 | 0.9498 | 0.0210 |
| 0.1 |  |  |  | -0.0947 | 3 | 0.9312 | 0.9147 | 0.0165 |
| 0 | 0.01 | 0.005 | 0.01 | -0.0948 | 4 | 0.8615 | 0.8683 | -0.0068 |
| 0.05 |  |  |  | -0.0923 | 4 | 0.8324 | 0.8453 | -0.0129 |
| 0.1 |  |  |  | -0.0848 | 5 | 0.9167 | 0.9154 | 0.0013 |
| 0 | 0.0225 | 0.005 | 0.01 | -0.0823 | 7 | 0.9022 | 0.8979 | 0.0043 |
| 0.05 |  |  |  | -0.0798 | 7 | 0.8703 | 0.8744 | -0.0041 |
| 0.1 |  |  |  | -0.0723 | 8 | 0.8489 | 0.8551 | -0.0062 |
| 0 | 0.0001 | 0.015 | 0.02 | -0.1047 | 4 | 0.8814 | 0.8725 | 0.0089 |
| 0.05 |  |  |  | -0.1022 | 4 | 0.8558 | 0.8554 | 0.0004 |
| 0.1 |  |  |  | -0.0947 | 5 | 0.9353 | 0.9256 | 0.0097 |
| 0 | 0.01 | 0.015 | 0.02 | -0.0948 | 6 | 0.9061 | 0.9012 | 0.0049 |
| 0.05 |  |  |  | -0.0923 | 6 | 0.8790 | 0.8794 | -0.0004 |
| 0.1 |  |  |  | -0.0848 | 7 | 0.8855 | 0.8764 | 0.0091 |
| 0 | 0.0225 | 0.015 | 0.02 | -0.0823 | 8 | 0.8049 | 0.8147 | -0.0098 |
| 0.05 |  |  |  | -0.0798 | 9 | 0.8415 | 0.8475 | -0.0060 |
| 0.1 |  |  |  | -0.0723 | 11 | 0.8482 | 0.8439 | 0.0043 |
| 0 | 0.0001 | 0.025 | 0.03 | -0.1047 | 6 | 0.9201 | 0.9042 | 0.0159 |
| 0.05 |  |  |  | -0.1022 | 6 | 0.8974 | 0.8900 | 0.0074 |
| 0.1 |  |  |  | -0.0947 | 6 | 0.8206 | 0.8191 | 0.0015 |
| 0 | 0.01 | 0.025 | 0.03 | -0.0948 | 7 | 0.8157 | 0.8213 | -0.0056 |
| 0.05 |  |  |  | -0.0923 | 8 | 0.8667 | 0.8647 | 0.0020 |
| 0.1 |  |  |  | -0.0848 | 9 | 0.8442 | 0.8436 | 0.0006 |
| 0 | 0.0225 | 0.025 | 0.03 | -0.0823 | 11 | 0.8384 | 0.8406 | -0.0022 |
| 0.05 |  |  |  | -0.0798 | 11 | 0.8024 | 0.8107 | -0.0083 |
| 0.1 |  |  |  | -0.0723 | 14 | 0.8258 | 0.8328 | -0.0070 |
