# Supplementary material for: Sample Size Determination for Individual Bioequivalence Inference
Source: PLoS One. 2014 Oct 13;9(10):e109746. doi: 10.1371/journal.pone.0109746 (PMC4195669; doi:10.1371/journal.pone.0109746)
Supplement: Table S2 — Sample size per sequence, asymptotical power, and empirical power for the linearized reference-scaled criterion with respect to a nominal power of 80% at the 5% significance level. (DOC) [file pone.0109746.s002.doc]

Table S2 Sample size per sequence, asymptotical power, and empirical power for the linearized reference-scaled criterion with respect to a nominal power of 80% at the 5% significance level

| $\delta$ | $\sigma_{D}^{2}$ | $\sigma_{WT}^{2}$ | $\sigma_{WR}^{2}$ | $\eta$ | $n$ | Asymptotic Power | Empirical Power | Difference  in Power |
| --- | --- | --- | --- | --- | --- | --- | --- | --- |
| 0 | 0.0001 | 0.04 | 0.04 | -0.0997 | 13 | 0.8186 | 0.8058 | 0.0128 |
| 0.05 |  |  |  | -0.0972 | 14 | 0.8208 | 0.8208 | 0.0000 |
| 0.1 |  |  |  | -0.0897 | 17 | 0.8165 | 0.8153 | 0.0012 |
| 0 | 0.01 | 0.04 | 0.04 | -0.0898 | 18 | 0.8203 | 0.8196 | 0.0007 |
| 0.05 |  |  |  | -0.0873 | 19 | 0.8127 | 0.8139 | -0.0012 |
| 0.1 |  |  |  | -0.0798 | 24 | 0.8149 | 0.8112 | 0.0037 |
| 0 | 0.0225 | 0.04 | 0.04 | -0.0773 | 27 | 0.8123 | 0.8144 | -0.0021 |
| 0.05 |  |  |  | -0.0748 | 29 | 0.8073 | 0.7994 | 0.0079 |
| 0.1 |  |  |  | -0.0673 | 37 | 0.8013 | 0.8027 | -0.0014 |
| 0 | 0.0001 | 0.09 | 0.09 | -0.2244 | 13 | 0.8192 | 0.8125 | 0.0067 |
| 0.05 |  |  |  | -0.2219 | 13 | 0.8079 | 0.8006 | 0.0073 |
| 0.1 |  |  |  | -0.2144 | 14 | 0.8021 | 0.7988 | 0.0033 |
| 0 | 0.01 | 0.09 | 0.09 | -0.2145 | 15 | 0.8200 | 0.8141 | 0.0059 |
| 0.05 |  |  |  | -0.2120 | 15 | 0.8082 | 0.8063 | 0.0019 |
| 0.1 |  |  |  | -0.2045 | 17 | 0.8196 | 0.8183 | 0.0013 |
| 0 | 0.0225 | 0.09 | 0.09 | -0.2020 | 18 | 0.8203 | 0.8177 | 0.0026 |
| 0.05 |  |  |  | -0.1995 | 18 | 0.8080 | 0.8004 | 0.0076 |
| 0.1 |  |  |  | -0.1920 | 20 | 0.8102 | 0.8076 | 0.0026 |
| 0 | 0.0001 | 0.16 | 0.16 | -0.3991 | 13 | 0.8195 | 0.8098 | 0.0097 |
| 0.05 |  |  |  | -0.3966 | 13 | 0.8131 | 0.7998 | 0.0133 |
| 0.1 |  |  |  | -0.3891 | 14 | 0.8217 | 0.8128 | 0.0089 |
| 0 | 0.01 | 0.16 | 0.16 | -0.3892 | 14 | 0.8176 | 0.8123 | 0.0053 |
| 0.05 |  |  |  | -0.3867 | 14 | 0.8111 | 0.8097 | 0.0014 |
| 0.1 |  |  |  | -0.3792 | 15 | 0.8174 | 0.8166 | 0.0008 |
| 0 | 0.0225 | 0.16 | 0.16 | -0.3767 | 15 | 0.8058 | 0.8062 | -0.0004 |
| 0.05 |  |  |  | -0.3742 | 16 | 0.8229 | 0.8138 | 0.0091 |
| 0.1 |  |  |  | -0.3667 | 16 | 0.8032 | 0.8013 | 0.0019 |
| 0 | 0.0001 | 0.25 | 0.25 | -0.6236 | 13 | 0.8196 | 0.8160 | 0.0036 |
| 0.05 |  |  |  | -0.6211 | 13 | 0.8155 | 0.8132 | 0.0023 |
| 0.1 |  |  |  | -0.6136 | 13 | 0.8033 | 0.8028 | 0.0005 |
| 0 | 0.01 | 0.25 | 0.25 | -0.6137 | 13 | 0.8005 | 0.7905 | 0.0100 |
| 0.05 |  |  |  | -0.6112 | 14 | 0.8241 | 0.8242 | -0.0001 |
| 0.1 |  |  |  | -0.6037 | 14 | 0.8119 | 0.8142 | -0.0023 |
| 0 | 0.0225 | 0.25 | 0.25 | -0.6012 | 14 | 0.8044 | 0.7960 | 0.0084 |
| 0.05 |  |  |  | -0.5987 | 15 | 0.8257 | 0.8200 | 0.0057 |
| 0.1 |  |  |  | -0.5912 | 15 | 0.8134 | 0.8142 | -0.0008 |

Table S2. Sample size per sequence, asymptotical power, and empirical power for the linearized reference-scaled criterion with respect to a nominal power of 80% at the 5% significance level (continued)

| $\delta$ | $\sigma_{D}^{2}$ | $\sigma_{WT}^{2}$ | $\sigma_{WR}^{2}$ | $\eta$ | $n$ | Asymptotic Power | Empirical Power | Difference  in Power |
| --- | --- | --- | --- | --- | --- | --- | --- | --- |
| 0 | 0.0001 | 0.06 | 0.04 | -0.0797 | 23 | 0.8084 | 0.8006 | 0.0078 |
| 0.05 |  | 0.06 | 0.04 | -0.0772 | 25 | 0.8088 | 0.8060 | 0.0028 |
| 0.1 |  | 0.06 | 0.04 | -0.0697 | 32 | 0.8068 | 0.8088 | -0.0020 |
| 0 | 0.01 | 0.06 | 0.04 | -0.0698 | 33 | 0.8092 | 0.8053 | 0.0039 |
| 0.05 |  | 0.06 | 0.04 | -0.0673 | 36 | 0.8072 | 0.8028 | 0.0044 |
| 0.1 |  | 0.06 | 0.04 | -0.0598 | 47 | 0.8003 | 0.7947 | 0.0056 |
| 0 | 0.0225 | 0.06 | 0.04 | -0.0573 | 54 | 0.8046 | 0.7978 | 0.0068 |
| 0.05 |  | 0.06 | 0.04 | -0.0548 | 60 | 0.8032 | 0.7940 | 0.0092 |
| 0.1 |  | 0.06 | 0.04 | -0.0473 | 84 | 0.8001 | 0.8013 | -0.0012 |
| 0 | 0.0001 | 0.11 | 0.09 | -0.2044 | 16 | 0.8016 | 0.7968 | 0.0048 |
| 0.05 |  | 0.11 | 0.09 | -0.2019 | 17 | 0.8119 | 0.8089 | 0.0030 |
| 0.1 |  | 0.11 | 0.09 | -0.1944 | 19 | 0.8167 | 0.8149 | 0.0018 |
| 0 | 0.01 | 0.11 | 0.09 | -0.1945 | 19 | 0.8113 | 0.8105 | 0.0008 |
| 0.05 |  | 0.11 | 0.09 | -0.1920 | 20 | 0.8174 | 0.8151 | 0.0023 |
| 0.1 |  | 0.11 | 0.09 | -0.1845 | 22 | 0.8148 | 0.8083 | 0.0065 |
| 0 | 0.0225 | 0.11 | 0.09 | -0.1820 | 23 | 0.8119 | 0.8068 | 0.0051 |
| 0.05 |  | 0.11 | 0.09 | -0.1795 | 24 | 0.8140 | 0.8184 | -0.0044 |
| 0.1 |  | 0.11 | 0.09 | -0.1720 | 26 | 0.8037 | 0.7964 | 0.0073 |
| 0 | 0.0001 | 0.18 | 0.16 | -0.3791 | 15 | 0.8199 | 0.8226 | -0.0027 |
| 0.05 |  | 0.18 | 0.16 | -0.3766 | 15 | 0.8133 | 0.8108 | 0.0025 |
| 0.1 |  | 0.18 | 0.16 | -0.3691 | 16 | 0.8175 | 0.8179 | -0.0004 |
| 0 | 0.01 | 0.18 | 0.16 | -0.3692 | 16 | 0.8142 | 0.8014 | 0.0128 |
| 0.05 |  | 0.18 | 0.16 | -0.3667 | 16 | 0.8073 | 0.7943 | 0.0130 |
| 0.1 |  | 0.18 | 0.16 | -0.3592 | 17 | 0.8097 | 0.8036 | 0.0061 |
| 0 | 0.0225 | 0.18 | 0.16 | -0.3567 | 18 | 0.8196 | 0.8133 | 0.0063 |
| 0.05 |  | 0.18 | 0.16 | -0.3542 | 18 | 0.8127 | 0.8062 | 0.0065 |
| 0.1 |  | 0.18 | 0.16 | -0.3467 | 19 | 0.8119 | 0.8119 | 0.0000 |
| 0 | 0.0001 | 0.27 | 0.25 | -0.6036 | 14 | 0.8136 | 0.8032 | 0.0104 |
| 0.05 |  | 0.27 | 0.25 | -0.6011 | 14 | 0.8094 | 0.8006 | 0.0088 |
| 0.1 |  | 0.27 | 0.25 | -0.5936 | 15 | 0.8224 | 0.8159 | 0.0065 |
| 0 | 0.01 | 0.27 | 0.25 | -0.5937 | 15 | 0.8202 | 0.8134 | 0.0068 |
| 0.05 |  | 0.27 | 0.25 | -0.5912 | 15 | 0.8160 | 0.8122 | 0.0038 |
| 0.1 |  | 0.27 | 0.25 | -0.5837 | 15 | 0.8034 | 0.7919 | 0.0115 |
| 0 | 0.0225 | 0.27 | 0.25 | -0.5812 | 16 | 0.8201 | 0.8181 | 0.0020 |
| 0.05 |  | 0.27 | 0.25 | -0.5787 | 16 | 0.8158 | 0.8157 | 0.0001 |
| 0.1 |  | 0.27 | 0.25 | -0.5712 | 16 | 0.8029 | 0.8050 | -0.0021 |

Table S2. Sample size per sequence, asymptotical power, and empirical power for the linearized reference-scaled criterion with respect to a nominal power of 80% at the 5% significance level (continued)

| $\delta$ | $\sigma_{D}^{2}$ | $\sigma_{WT}^{2}$ | $\sigma_{WR}^{2}$ | $\eta$ | $n$ | Asymptotic Power | Empirical Power | Difference  in Power |
| --- | --- | --- | --- | --- | --- | --- | --- | --- |
| 0 | 0.0001 | 0.02 | 0.04 | -0.1197 | 8 | 0.8356 | 0.8275 | 0.0081 |
| 0.05 |  |  |  | -0.1172 | 8 | 0.8137 | 0.8118 | 0.0019 |
| 0.1 |  |  |  | -0.1097 | 10 | 0.8329 | 0.8289 | 0.0040 |
| 0 | 0.01 | 0.02 | 0.04 | -0.1098 | 10 | 0.8012 | 0.8051 | -0.0039 |
| 0.05 |  |  |  | -0.1073 | 11 | 0.8147 | 0.8121 | 0.0026 |
| 0.1 |  |  |  | -0.0998 | 13 | 0.8074 | 0.8089 | -0.0015 |
| 0 | 0.0225 | 0.02 | 0.04 | -0.0973 | 15 | 0.8072 | 0.8034 | 0.0038 |
| 0.05 |  |  |  | -0.0948 | 16 | 0.8054 | 0.8000 | 0.0054 |
| 0.1 |  |  |  | -0.0873 | 20 | 0.8107 | 0.8067 | 0.0040 |
| 0 | 0.0001 | 0.07 | 0.09 | -0.2444 | 10 | 0.8114 | 0.8127 | -0.0013 |
| 0.05 |  |  |  | -0.2419 | 10 | 0.8005 | 0.7953 | 0.0052 |
| 0.1 |  |  |  | -0.2344 | 11 | 0.8057 | 0.8101 | -0.0044 |
| 0 | 0.01 | 0.07 | 0.09 | -0.2345 | 12 | 0.8280 | 0.8238 | 0.0042 |
| 0.05 |  |  |  | -0.2320 | 12 | 0.8170 | 0.8107 | 0.0063 |
| 0.1 |  |  |  | -0.2245 | 13 | 0.8146 | 0.8044 | 0.0102 |
| 0 | 0.0225 | 0.07 | 0.09 | -0.2220 | 14 | 0.8197 | 0.8125 | 0.0072 |
| 0.05 |  |  |  | -0.2195 | 14 | 0.8081 | 0.8093 | -0.0012 |
| 0.1 |  |  |  | -0.2120 | 16 | 0.8237 | 0.8176 | 0.0061 |
| 0 | 0.0001 | 0.14 | 0.16 | -0.4191 | 11 | 0.8082 | 0.8036 | 0.0046 |
| 0.05 |  |  |  | -0.4166 | 11 | 0.8019 | 0.8002 | 0.0017 |
| 0.1 |  |  |  | -0.4091 | 12 | 0.8167 | 0.8068 | 0.0099 |
| 0 | 0.01 | 0.14 | 0.16 | -0.4092 | 12 | 0.8117 | 0.8008 | 0.0109 |
| 0.05 |  |  |  | -0.4067 | 12 | 0.8052 | 0.7947 | 0.0105 |
| 0.1 |  |  |  | -0.3992 | 13 | 0.8168 | 0.8160 | 0.0008 |
| 0 | 0.0225 | 0.14 | 0.16 | -0.3967 | 13 | 0.8043 | 0.7963 | 0.0080 |
| 0.05 |  |  |  | -0.3942 | 14 | 0.8256 | 0.8259 | -0.0003 |
| 0.1 |  |  |  | -0.3867 | 14 | 0.8066 | 0.7984 | 0.0082 |
| 0 | 0.0001 | 0.23 | 0.25 | -0.6436 | 12 | 0.8227 | 0.8186 | 0.0041 |
| 0.05 |  |  |  | -0.6411 | 12 | 0.8187 | 0.8099 | 0.0088 |
| 0.1 |  |  |  | -0.6336 | 12 | 0.8068 | 0.8018 | 0.0050 |
| 0 | 0.01 | 0.23 | 0.25 | -0.6337 | 12 | 0.8037 | 0.8008 | 0.0029 |
| 0.05 |  |  |  | -0.6312 | 13 | 0.8296 | 0.8264 | 0.0032 |
| 0.1 |  |  |  | -0.6237 | 13 | 0.8178 | 0.8108 | 0.0070 |
| 0 | 0.0225 | 0.23 | 0.25 | -0.6212 | 13 | 0.8101 | 0.8012 | 0.0089 |
| 0.05 |  |  |  | -0.6187 | 13 | 0.8059 | 0.7996 | 0.0063 |
| 0.1 |  |  |  | -0.6112 | 14 | 0.8214 | 0.8187 | 0.0027 |
