# Supplementary material for: Sample Size Determination for Individual Bioequivalence Inference
Source: PLoS One. 2014 Oct 13;9(10):e109746. doi: 10.1371/journal.pone.0109746 (PMC4195669; doi:10.1371/journal.pone.0109746)
Supplement: Table S3 — Comparison of sample sizes required between the 2×3 and 2×4 crossover designs with respect to a nominal power of 80% at the 5% significance level. (DOC) [file pone.0109746.s003.doc]

Table S3 Comparison of sample sizes required between the 2x3 and 2x4 crossover designs with respect to a nominal power of 80% at the 5% significance level

|  |  |  |  | 2×3 crossover design | | | 2×4 crossover design | | | |
| --- | --- | --- | --- | --- | --- | --- | --- | --- | --- | --- |
| $\sigma_{WT}^{2}=\sigma_{WR}^{2}$ | $\sigma_{D}^{2}$ | $\eta$ | *n* | Asymptotic Power | Empirical Power | Difference  in Power | *n* | Asymptotic Power | Empirical Power | Difference  in Power |
| 0. 0225 | 0.0001 | -0.09719 | 9 | 0.8695 | 0.8795 | -0.0100 | 5 | 0.9407 | 0.9215 | 0.0192 |
|  | 0.01 | -0.08729 | 12 | 0.8476 | 0.8652 | -0.0176 | 7 | 0.8263 | 0.8313 | -0.0050 |
|  | 0.0225 | -0.07479 | 17 | 0.8067 | 0.8341 | -0.0274 | 11 | 0.8607 | 0.8639 | -0.0032 |
| 0.0529 | 0.0001 | -0.12937 | 28 | 0.8118 | 0.8121 | -0.0003 | 14 | 0.8094 | 0.8050 | 0.0044 |
|  | 0.01 | -0.11947 | 35 | 0.8097 | 0.8123 | -0.0026 | 17 | 0.8021 | 0.8036 | -0.0015 |
|  | 0.0225 | -0.10697 | 47 | 0.8068 | 0.8090 | -0.0022 | 23 | 0.8102 | 0.8101 | 0.0001 |
| 0.09 | 0.0001 | -0.22193 | 27 | 0.8080 | 0.8116 | -0.0036 | 13 | 0.8010 | 0.7967 | 0.0043 |
|  | 0.01 | -0.21203 | 31 | 0.8099 | 0.8137 | -0.0038 | 15 | 0.8059 | 0.8015 | 0.0044 |
|  | 0.0225 | -0.19953 | 36 | 0.8021 | 0.7994 | 0.0027 | 18 | 0.8107 | 0.8042 | 0.0065 |
| 0.25 | 0.0001 | -0.62110 | 26 | 0.8031 | 0.7947 | 0.0084 | 13 | 0.8237 | 0.8241 | -0.0004 |
|  | 0.01 | -0.61120 | 28 | 0.8126 | 0.8155 | -0.0029 | 14 | 0.8048 | 0.7985 | 0.0063 |
|  | 0.0225 | -0.59870 | 29 | 0.8038 | 0.8031 | 0.0007 | 14 | 0.8086 | 0.8079 | 0.0007 |
