# Supplementary material for: Sample Size Determination for Individual Bioequivalence Inference
Source: PLoS One. 2014 Oct 13;9(10):e109746. doi: 10.1371/journal.pone.0109746 (PMC4195669; doi:10.1371/journal.pone.0109746)
Supplement: Table S4 — Comparison between different methods for determining the scaled criterion with respect to a nominal power of 80% at the 5% significance level, where , , and . (DOC) [file pone.0109746.s004.doc]

Table S4. Comparison between different methods for determining the scaled criterion with respect to a nominal power of 80% at the 5% significance level, where $\delta=0.1$, $\sigma_{D}^{2}=0.0225$, and $\sigma_{WT}^{2}=\sigma_{WR}^{2}$.

|  |  |  | Difference in Asymptotic Power - Empirical Power | | |
| --- | --- | --- | --- | --- | --- |
| $\sigma_{WR}^{2}$ | $\eta$ | $n$ | EST | TEST | OPT |
| 0.020 | -0.0673 | 12 | -0.0028 | -0.0046 | -0.0157 |
| 0.025 | -0.0673 | 14 | 0.0021 | -0.0062 | -0.0040 |
| 0.030 | -0.0673 | 16 | 0.0006 | -0.0030 | -0.0102 |
| 0.035 | -0.0673 | 18 | 0.0137 | -0.0099 | -0.0110 |
| 0.040 | -0.0673 | 37 | -0.1614 | -0.0386 | 0.0086 |
| 0.045 | -0.0798 | 33 | -0.1167 | -0.0018 | 0.0024 |
| 0.050 | -0.0922 | 29 | -0.0683 | -0.0044 | 0.0104 |
| 0.055 | -0.1047 | 27 | -0.0287 | 0.0038 | 0.0066 |
| 0.060 | -0.1172 | 25 | -0.0071 | 0.0020 | 0.0031 |
| 0.065 | -0.1297 | 24 | -0.0078 | 0.0058 | 0.0009 |
| 0.070 | -0.1421 | 23 | 0.0028 | 0.0031 | 0.0022 |
| 0.075 | -0.1546 | 22 | 0.0017 | -0.0008 | 0.0038 |
| 0.080 | -0.1671 | 21 | 0.0004 | -0.0035 | 0.0041 |

EST: Estimation method suggested by Hyslop, et al. [7]

TEST: Test method at the significance level of 0.05, Chow, et al [8]

OPT: Assuming that we know whether $\sigma_{WR}^{2}\geq\sigma_{W0}^{2}$, Chow, et al. [8]
