# Supplementary material for: Sample Size Determination for Individual Bioequivalence Inference
Source: PLoS One. 2014 Oct 13;9(10):e109746. doi: 10.1371/journal.pone.0109746 (PMC4195669; doi:10.1371/journal.pone.0109746)
Supplement: File S1 — Derivation of the upper confidence bound under 2×4 crossover design by the modified large sample method. (DOC) [file pone.0109746.s005.doc]

**Supporting Information S1 Derivation of the 100(1-α)% upper confidence bound under 2×4 crossover design by the modified large sample method**

Consider the following two-sequence and four-period (2×4) crossover design for evaluation of IBE:

|  | Period | | | |
| --- | --- | --- | --- | --- |
| Sequence | I | II | III | IV |
| 1 | T | R | T | R |
| 2 | R | T | R | T |

where T: test (generic) formulation; R: reference (innovative) formulation. Let $Y_{ijkl}$ denote the logarithmic transformation of the pharmacokinetic (PK) response of the *l*th repeated administration of formulation *k* for subject *j* in sequence *i*, where $i=1,2$, $j=1,2,...,n$, $k=R,T$ and $l=1,2$. The model with an assumption of no carryover effects is given as follows:

$Y_{ijkl}=\mu_{k}+\gamma_{ikl}+\zeta_{ijk}+\varepsilon_{ijkl}$ (S1.1)

where $\mu_{k}$ is the overall mean for the formulation. The term $\gamma_{ikl}$ represents the fixed effect of replicate *l* on formulation *k* in sequence *i*; $\zeta_{ijk}$is the random subject effect for subject *j* in sequence *i* receiving formulation *k*; and $\varepsilon_{ijkl}$is the random error associated with the *l*th replicated administration of formulation *k* received by subject *j* in sequence *i*.

To avoid over-parameterization of the model, the following constraints are put on the nuisance parameters $\gamma_{ikl}$:

$$\sum_{i=1}^{2} \sum_{l=1}^{2} \gamma_{ikl}=0,\text{ for }k=T\text{ and }R.$$

The bivariate vector of random subject effects $\mathbf{\zeta}_{ij}=(\zeta_{ijT},\zeta_{ijR})'$ is also assumed to be mutually independent and distributed between subjects as a bivariate normal random vector with mean vector $\mathbf{0}$ and covariance matrix

$\mathbf{\Sigma}_{\mathbf{B}}=\left(\begin{matrix} \sigma_{BR}^{2} & \rho\sigma_{BR}\sigma_{BT} \\ \rho\sigma_{BR}\sigma_{BT} & \sigma_{BT}^{2} \end{matrix}\right)$, $j=1,\ldots,n$, $i=1,2$.

The random error $\varepsilon_{ijkl}$ is assumed to be mutually independent and identically distributed as $N(0,\sigma_{Wk}^{2})$ for subject *j* in sequence *i* on formulation *k*. In addition, bivariate vectors of random subject effects are also independent of random errors. The parameters of interest are $\delta=\mu_{T}-\mu_{R}$, $\sigma_{WT}^{2}$, $\sigma_{WR}^{2}$ and

$\sigma_{D}^{2}=\sigma_{BT}^{2}+\sigma_{BR}^{2}-2\rho\sigma_{BT}\sigma_{BR}$.

Define the following mutually independent within-subject linear contrasts:

$I_{ij}=\frac{1}{2}\left[\left(Y_{ijT1}+Y_{ijT2}\right)-\left(Y_{ijR1}+Y_{ijR2}\right)\right]$,

$T_{ij}=(Y_{ijT1}-Y_{ijT2})/\sqrt{2}$,

$R_{ij}=Y_{ijR1}-Y_{ijR2}/\sqrt{2}$, $i=1,2$, $j=1,2,...,n$.

Their respective expected values and variances are given as

$E(I_{ij})=\mu_{T}-\mu_{R}$, $V(I_{ij})=\sigma_{0.5,0.5}^{2}=\sigma_{D}^{2}+\frac{1}{2}(\sigma_{WR}^{2}+\sigma_{WT}^{2})$,

$E(T_{ij})=E(R_{ij})=0$, $V(T_{ij})=\sigma_{WT}^{2}$ and $V(R_{ij})=\sigma_{WR}^{2}$.

Consequently, under the above-mentioned 2×4 crossover design, the linearized criterion can be re-expressed in term of $\text{σ}_{\text{0.5,0.5}}^{2}$as

(S1.2)

The reformulation of in Equation (S1.2) can avoid direct estimation of $\sigma_{D}^{2}$ through $\sigma_{BT}^{2}$, $\sigma_{BR}^{2}$ and $\rho$.

Then unbiased estimators for each individual component in *η* are given as

$\hat{\delta}=\hat{\mu}_{T}-\hat{\mu}_{R}=\frac{1}{2n}\sum_{i=1}^{2} \sum_{j=1}^{n} I_{ij}=\frac{1}{N}\sum_{i=1}^{2} \sum_{j=1}^{n} I_{ij}$,

$\hat{\sigma}_{I}^{2}=\hat{\sigma}_{0.5,0.5}^{2}=\frac{1}{2(n-1)}\sum_{i=1}^{2} \sum_{j=1}^{n} \left(I_{ij}-\bar{I}_{i}\right)^{2}=\frac{1}{N-2}\sum_{i=1}^{2} \sum_{j=1}^{n} \left(I_{ij}-\bar{I}_{i}\right)^{2}$,

$\hat{\sigma}_{WT}^{2}=\frac{1}{2(n-1)}\sum_{i=1}^{2} \sum_{j=1}^{n} \left(T_{ij}-\bar{T}_{i}\right)^{2}=\frac{1}{N-2}\sum_{i=1}^{2} \sum_{j=1}^{n} \left(T_{ij}-\bar{T}_{i}\right)^{2}$,

$\hat{\sigma}_{WR}^{2}=\frac{1}{2(n-1)}\sum_{i=1}^{2} \sum_{j=1}^{n} \left(R_{ij}-\bar{R}_{i}\right)^{2}=\frac{1}{N-2}\sum_{i=1}^{2} \sum_{j=1}^{n} \left(R_{ij}-\bar{R}_{i}\right)^{2}$.

where $\bar{I}_{i}=\sum_{j=1}^{n} I_{ij}/n$, $\bar{T}_{i}=\sum_{j=1}^{n} T_{ij}/n$,$\bar{R}_{i}=\sum_{j=1}^{n} R_{ij}/n$, and $N=2n$. In addition, $\hat{\delta}$, $\hat{\sigma}_{0.5,0.5}^{2}$, $\hat{\sigma}_{WT}^{2}$, and $\hat{\sigma}_{WR}^{2}$ are mutually independent. Consequently, the MLS $100(1-\alpha)\%$ upper confidence bound proposed by Hyslop et al [7] is given as

$\hat{\tau}=\hat{\eta}\text{ + }\sqrt{U}$, (S1.3)

where , and

. (S1.4)

with if $\sigma_{WR}^{2}\geq\sigma_{W0}^{2}$ and if $\sigma_{WR}^{2}<\sigma_{W0}^{2}$. The null hypothesis is rejected and the IBE is concluded at the $\alpha$ significance level if the MLS $100(1-\alpha)\%$ upper confidence bound given in Equation (S1.3) is less than zero.
