# Supplementary material for: Sample Size Determination for Individual Bioequivalence Inference
Source: PLoS One. 2014 Oct 13;9(10):e109746. doi: 10.1371/journal.pone.0109746 (PMC4195669; doi:10.1371/journal.pone.0109746)
Supplement: File S2 — Derivation of the asymptotic normality of the upper confidence bound. (DOC) [file pone.0109746.s006.doc]

Supporting Information S2 Derivation of the asymptotic normality of the upper confidence bound

The estimators $\hat{\delta}^{2}$, $\hat{\sigma}_{0.5,0.5}^{2}$, $\hat{\sigma}_{WT}^{2}$, and $\hat{\sigma}_{WR}^{2}$ are either the first sample moment or the second sample moments which are also mutually independent of each other. Either by delta method or by the projection technique of U-statistics [9], it follows that the vector $(\hat{\delta}^{2},\hat{\sigma}_{0.5,0.5}^{2},\hat{\sigma}_{WT}^{2},\hat{\sigma}_{WR}^{2})'$ is asymptotically distributed as a multivariate normal distribution with mean vector $(\delta^{2},\sigma_{0.5,0.5}^{2},\sigma_{WT}^{2},\sigma_{WR}^{2})'$ and covariance matrix

$\frac{1}{N}\left(\begin{matrix} 4\delta^{2}\sigma_{0.5,0.5}^{2}\text{ 0 0 0 } \\ \text{0 2}\sigma_{0.5,0.5}^{4}\text{ 0 0} \\ \text{0 0 2}\sigma_{WT}^{4}\text{ 0} \\ \text{0 0 0 2}\sigma_{WR}^{4} \end{matrix}\right)$,

where $N=2n$.

To construct the asymptotic distribution of the MLS 100(1-α)% upper confidence bound, it is necessary to derive the mean and variance of each estimator in the upper confidence bound. The random variable $\hat{\delta}^{2}/(\sigma_{0.5,0.5}^{2}/2n)$ is distributed as noncentral chi-square with 1 degree of freedom and the noncentral parameter $2n\delta^{2}/\sigma_{0.5,0.5}^{2}$. Thus the mean and variance of $\hat{\delta}^{2}$ are, respectively,

$\delta^{2}+\frac{\sigma_{0.5,0.5}^{2}}{2n}$ and $\frac{2\delta^{2}\sigma_{0.5,0.5}^{2}}{n}+\frac{\sigma_{0.5,0.5}^{4}}{2n^{2}}$.

The distributions of the other estimators for each individual variance component in *η* are given as

$\hat{\sigma}_{h}^{2}\sim\frac{\chi_{2(n-1)}^{2}\sigma_{h}^{2}}{2(n-1)}$, $h=(0.5,0.5),\text{ }WT,\text{ }WR$,

where $\chi_{r}^{2}$ denotes a central chi-square distribution with $r$ degrees of freedom. Hence the mean and variance of $\hat{\sigma}_{h}^{2}$ are $\sigma_{h}^{2}$ and $\sigma_{h}^{4}/(n-1)$, respectively.

For a better approximation to the asymptotic normal distribution of the MLS 100(1-α)% upper bound for sample size determination in IBE evaluation, we apply a stochastic expansion, suggested by Barndorff-Nielsen and Cox [10], of random variables $\sum_{i=1}^{n} Y_{n}$ in terms of the standard normal variables as

$\left[\sum_{i=1}^{n} Y_{n}-\mu\left(\sum_{i=1}^{n} Y_{n}\right)\right]/\sigma\left(\sum_{i=1}^{n} Y_{n}\right)=Z+(Z^{2}-1)\rho_{3}\left(\sum_{i=1}^{n} Y_{n}\right)/6+...$, (S2.1)

where $Z$ denotes a standard normal random variable and $\mu(\cdot)$, $\sigma(\cdot)$ and $\rho_{3}(\cdot)$ are the mean, the standard deviation, and the skewness of $\sum_{i=1}^{n} Y_{n}$, respectively. For simplicity, denote $X_{1}=\hat{\delta}^{2}$, $X_{2}=\hat{\sigma}_{0.5,0.5}^{2}$, $X_{3}=\hat{\sigma}_{WT}^{2}$, and $X_{4}=\hat{\sigma}_{WR}^{2}$ with their corresponding parameters $\theta_{1}=\delta^{2}+\sigma_{0.5,0.5}^{2}/(2n)$, $\theta_{2}=\sigma_{0.5,0.5}^{2}$, $\theta_{3}=\sigma_{WT}^{2}$, $\theta_{4}=\sigma_{WR}^{2}$. Then the estimators in the upper confidence bound can be expressed in terms of the standard normal variables according to (S2.1) as

$\left(X_{h}-\theta_{h}\right)/\sigma(X_{h})=Z_{h}+(Z_{h}^{2}-1)\rho_{3}(X_{h})/6+...$, $h=1,2,3,4$. (S2.2)

Moreover, denote

$t=t_{1-\alpha,2(n-1)}$, $q_{1}=\left(\frac{2(n-1)}{\chi_{\alpha,2(n-1)}^{2}}-1\right)^{2}$, $q_{2}=\left(\frac{2(n-1)}{\chi_{1-\alpha,2(n-1)}^{2}}-1\right)^{2}$,

$\mathbf{X}=(X_{1},X_{2},X_{3},X_{4})'$, and $\mathbf{\theta}=(\theta_{1},\theta_{2},\theta_{3},\theta_{4})'$.

Here $t_{p,r}$ and $\chi_{p,r}^{2}$ are the $100p$th percentiles of the central t and central chi-square distribution with $r$ degrees of freedom. Then the $100(1-\alpha)\%$ upper confidence bound for $\eta$ under the 2×4 crossover design can be represented by a function $g(\cdot)$ as

$g(x_{1},x_{2},x_{3},x_{4})\text{ }=\text{ }x_{1}+x_{2}+c_{3}x_{3}+c_{4}x_{4}+c+\sqrt{u(x_{1},x_{2},x_{3},x_{4})}$, (S2.3)

where

$$u(x_{1},x_{2},x_{3},x_{4})=\left[\left(\sqrt{x_{1}}+\frac{t}{\sqrt{2n}}x_{2}^{1/2}\right)^{2}-x_{1}\right]^{2}+d_{2}x_{2}^{2}+d_{3}x_{3}^{2}+d_{4}x_{4}^{2}$$

$=\frac{2^{1/2}t^{3}}{n^{3/2}}x_{1}^{1/2}x_{2}^{3/2}+\frac{2t^{2}}{n}x_{1}x_{2}+\left(d_{2}+\frac{t^{4}}{4n^{2}}\right)x_{2}^{2}+d_{3}x_{3}^{2}+d_{4}x_{4}^{2}$,

with constants $c_{3}=1/2$, , , $d_{2}=q_{1}$, $d_{3}=q_{1}/4$, . Then, the partial derivatives of $g(\cdot)$with respect to **x**, evaluated at $\mathbf{\theta}$ are given as

$$g_{1}=\frac{\partial}{\partial x_{1}}g(\mathbf{x})|_{\mathbf{x}=\mathbf{\theta}}=1+u^{-1/2}(\mathbf{\theta})\left[\frac{t^{3}}{(2n)^{3/2}}\left(\delta^{2}+\frac{\sigma_{0.5,0.5}^{2}}{2n}\right)^{-1/2}\left(\sigma_{0.5,0.5}^{2}\right)^{3/2}+\frac{t^{2}}{n}\sigma_{0.5,0.5}^{2}\right]$$

$$g_{2}=\frac{\partial}{\partial x_{2}}g(\mathbf{x})|_{\mathbf{x}=\mathbf{\theta}}$$

$$=1+u^{-1/2}(\mathbf{\theta})\left[\begin{matrix} \frac{3t^{3}}{(2n)^{3/2}}\left(\delta^{2}+\frac{\sigma_{0.5,0.5}^{2}}{2n}\right)^{1/2}\left(\sigma_{0.5,0.5}^{2}\right)^{1/2} \\ +\frac{t^{2}}{n}\left(\delta^{2}+\frac{\sigma_{0.5,0.5}^{2}}{2n}\right)+\left(d_{2}+\frac{t^{4}}{4n^{2}}\right)\sigma_{0.5,0.5}^{2} \end{matrix}\right]$$

$g_{3}=\frac{\partial}{\partial x_{3}}g(\mathbf{x})|_{\mathbf{x}=\mathbf{\theta}}=c_{3}+u^{-1/2}(\mathbf{\theta})d_{3}\sigma_{WT}^{2}$,

$g_{4}=\frac{\partial}{\partial x_{4}}g(\mathbf{x})|_{\mathbf{x}=\mathbf{\theta}}=c_{4}+u^{-1/2}(\mathbf{\theta})d_{4}\sigma_{WR}^{2}$.

Then the Taylor expansion of $g(\mathbf{X})$ with respect to the random vector $\mathbf{X}$ at $\mathbf{\theta}$ is given as

$$g(\mathbf{X})=g(\mathbf{\theta})+\sum_{h=1}^{4} g_{h}\times\left(X_{h}-\theta_{h}\right)+...$$

$=g(\mathbf{\theta})+\sum_{h=1}^{4} g_{h}\times\left[\sigma(X_{h})Z_{h}+(Z_{h}^{2}-1)\sigma(X_{h})\rho_{3}(X_{h})/6+...\right]+...$, (S2.4)

where the term $\sum_{h=1}^{4} g_{h}\times\left[(Z^{2}-1)\sigma(X_{h})\rho_{3}(X_{h})/6\right]+...$ has an order of $n^{-1}$ and converges to zero as $n$ becomes sufficiently large. It follows from (S2.4) that $\hat{\tau}$ is asymptotically normal with mean $\mu_{\hat{\tau}}$ and variance $\sigma_{\hat{\tau}}$, where

, (S2.5)

and

$\sigma_{\hat{\tau}}^{2}=g_{1}^{2}\left(\frac{2\delta^{2}\sigma_{0.5,0.5}^{2}}{n}+\frac{\sigma_{0.5,0.5}^{4}}{2n^{2}}\right)+\frac{1}{n-1}\left(g_{2}^{2}\sigma_{0.5,0.5}^{4}+g_{3}^{2}\sigma_{WT}^{4}+g_{4}^{2}\sigma_{WR}^{4}\right)$ (S2.6)

with

$$\begin{matrix} u(\mathbf{\theta})=\frac{2^{1/2}t^{3}}{n^{3/2}}\left(\delta^{2}+\frac{\sigma_{0.5,0.5}^{2}}{2n}\right)^{1/2}\left(\sigma_{0.5,0.5}^{2}\right)^{3/2}+\frac{2t^{2}}{n}\left(\delta^{2}+\frac{\sigma_{0.5,0.5}^{2}}{2n}\right)\sigma_{0.5,0.5}^{2} \\ \text{ }+\left(d_{2}+\frac{t^{4}}{4n^{2}}\right)\sigma_{0.5,0.5}^{4}+d_{3}\sigma_{WT}^{4}+d_{4}\sigma_{WR}^{4} \end{matrix}$$
