# Supplementary material for: Sample Size Determination for Individual Bioequivalence Inference
Source: PLoS One. 2014 Oct 13;9(10):e109746. doi: 10.1371/journal.pone.0109746 (PMC4195669; doi:10.1371/journal.pone.0109746)
Supplement: File S3 — SAS macro code. (DOC) [file pone.0109746.s007.doc]

**Supporting Information S3 SAS Macro Codes**

/*----------------------------------Parameters setting------------------------------*/

/* Users should provide the following specifications of the parameters for sample size determination */

%let delta=0.05; /* The difference in averages */

%let sigma_d=0.0001; /* The variance of formulation-by-subject interaction */

%let sigma_wt=0.0225; /* The within-subject variance for test formulation */

%let sigma_wr=0.0225; /* The within-subject variance for reference formulation */

%let sigma0=0.04; /* The predefined upper limit for the within-subject variance */

%let theta0=2.4948; /* The equivalence limit of the IBE criterion for reference formulation */

%let alpha=0.05; /* The significance level */

%let power=0.8; /* The required power */

/*----------------------------------------------------------------------------------*/

**%macro** ***nlp***;

proc nlp outest=aaa noprint;

min total_n;

decvar n=**2**;

bounds n>=**2**;

nlincon eq=**0**;

delta=&delta;

sigma_d=&sigma_d;

sigma_wt=&sigma_wt;

sigma_wr=&sigma_wr;

if sigma_wr>=&sigma0 then phi=**1**; else if sigma_wr<&sigma0 then phi=**0**;

sigma_i=sigma_d+(sigma_wt+sigma_wr)/**2**;

c3=**1**/**2**;

c4=-**3**/**2**-phi*&theta0;

c=&theta0*(phi-**1**)*&sigma0;

t=quantile("t",**1**-&alpha,**2***(n-**1**));

d2=(**2***(n-**1**)/quantile("chisq", &alpha, **2***(n-**1**))-**1**)****2**;

d3=(c3*(**2***(n-**1**)/quantile("chisq", &alpha, **2***(n-**1**))-**1**))****2**;

d4=(c4*(**2***(n-**1**)/quantile("chisq", **1**-&alpha, **2***(n-**1**))-**1**))****2**;

thetad=(delta****2**+sigma_i/(**2***n));

eta=delta****2**+sigma_i+c3*sigma_wt+c4*sigma_wr+c;

u=**2****(**1**/**2**)*t****3**/n**(**3**/**2**)*thetad**(**1**/**2**)*sigma_i**(**3**/**2**)+**2***t****2**/n*thetad*sigma_i+(d2+t****4**/(**4***n****2**))*sigma_i****2**+d3*sigma_wt****2**+d4*sigma_wr****2**;

g1=**1**+u**(-**1**/**2**)*(t****3**/(**2***n)**(**3**/**2**)*thetad**(-**1**/**2**)*sigma_i**(**3**/**2**)+t****2***sigma_i/n);

g2=**1**+u**(-**1**/**2**)*(**3***t****3**/(**2***n)**(**3**/**2**)*thetad**(**1**/**2**)*sigma_i**(**1**/**2**)+t****2**/n*thetad+(d2+t****4**/(**4***n****2**))*sigma_i);

g3=c3+u**(-**1**/**2**)*d3*sigma_wt;

g4=c4+u**(-**1**/**2**)*d4*sigma_wr;

mu=thetad+sigma_i+c3*sigma_wt+c4*sigma_wr+c+sqrt(u);

sigma=g1****2***(**2***delta****2***sigma_i/n+sigma_i****2**/(**2***n****2**))+(g2****2***sigma_i****2**+g3****2***sigma_wt****2**+g4****2***sigma_wr****2**)/(n-**1**);

eq=mu+quantile("normal", &power)*sqrt(sigma);

total_n=n;

run;

data bbb; set aaa; if _type_="PARMS"; n=ceil(n); drop _tech_ _type_ _name_ _rhs_ _iter_;

run;

ods html; ods escapechar='^';

proc print noobs ;

%if &sigma_wr < &sigma0 %then %do; %let scaled=constant; %end;

%else %if &sigma_wr >= &sigma0 %then %do; %let scaled=reference; %end;

title h=**1** "The sample size per sequence determined by the &scaled.-scaled criterion with

the significance level &alpha and power &power";

footnote h=**1** "The alternative parameters are given as δ = &delta., σ^{super 2}^{sub D} = &sigma_d, σ^{super 2}^{sub WT} = &sigma_wt, σ^{super 2}^{sub WR} = &sigma_wr..";

footnote2 h=**1** "The upper limit of the IBE criterion is θ^{sub 0} = &theta0..";

footnote3 h=**1** "The predefined upper limit for the within-subject variance is σ^{super 2}^{sub W0} = &sigma0..";

run; ods html close;

**%mend**;

%***nlp***

**The output for an example**

**
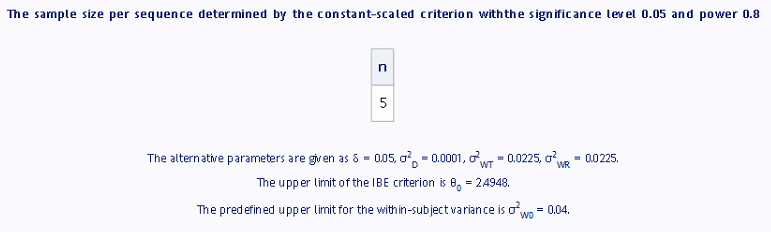
**
